# The role of natural killer cells in Parkinson’s disease

**DOI:** 10.1038/s12276-020-00505-7

**Published:** 2020-09-24

**Authors:** Rachael H. Earls, Jae-Kyung Lee

**Affiliations:** Department of Physiology and Pharmacology, University of Georgia College of Veterinary Medicine, Athens, GA 30602 USA

**Keywords:** Neuroimmunology, Neuroimmunology

## Abstract

Numerous lines of evidence indicate an association between sustained inflammation and Parkinson’s disease, but whether increased inflammation is a cause or consequence of Parkinson’s disease remains highly contested. Extensive efforts have been made to characterize microglial function in Parkinson’s disease, but the role of peripheral immune cells is less understood. Natural killer cells are innate effector lymphocytes that primarily target and kill malignant cells. Recent scientific discoveries have unveiled numerous novel functions of natural killer cells, such as resolving inflammation, forming immunological memory, and modulating antigen-presenting cell function. Furthermore, natural killer cells are capable of homing to the central nervous system in neurological disorders that exhibit exacerbated inflammation and inhibit hyperactivated microglia. Recently, a study demonstrated that natural killer cells scavenge alpha-synuclein aggregates, the primary component of Lewy bodies, and systemic depletion of natural killer cells results in exacerbated neuropathology in a mouse model of alpha-synucleinopathy, making them a highly relevant cell type in Parkinson’s disease. However, the exact role of natural killer cells in Parkinson’s disease remains elusive. In this review, we introduce the systemic inflammatory process seen in Parkinson’s disease, with a particular focus on the direct and indirect modulatory capacity of natural killer cells in the context of Parkinson’s disease.

## Parkinson’s disease: How natural killer cells can help

Understanding the roles that inflammation and immune cells called natural killer (NK) cells play in Parkinson’s disease (PD) may help in finding new treatments. In PD, a neurodegenerative disease, the protein α-synuclein is misshapen and accumulates in brain cells, causing inflammation. NK cells, which mostly target cancer cells, have recently been shown to resolve inflammation. Jae-Kyung Lee and Rachael H. Earls of the University of Georgia College of Veterinary Medicine, Athens, USA, reviewed the role of NK cells in PD. They report that NK cells can degrade α-synuclein aggregates. Further, NK cells are recruited to areas of inflammation where they then decrease the α-synuclein burden and reduce inflammation. Although further research is needed to understand how age and PD affect NK cell number and functions, these results may illuminate pathways to better treatments for PD.

## Introduction

Parkinson’s disease (PD) is the second most common neurodegenerative disease that affects over one million people in the United States, with predictable healthcare-associated costs of nearly $52 billion per year^1^. PD is pathologically characterized by the misfolding and aggregation of the alpha-synuclein (α-syn) protein into insoluble inclusions known as Lewy bodies (LBs) and Lewy neurites^2^. Monomeric α-syn protein is highly expressed in presynaptic terminals^3^ and the nucleus^4^ of cells. However, α-syn can acquire toxic properties through the pathogenic process of misfolding into α-syn fibrils that comprise the major component of LBs. LBs are present in PD, dementia with Lewy bodies (DLB), and Alzheimer’s disease (AD) brains; however, each disease shows distinct regional specificity, with PD patients displaying LBs most prominently in the substantia nigra (SN)^5^. Furthermore, in human sporadic PD cases and murine models of PD, LB formation is present in numerous cell types other than dopaminergic (DA) neurons such as astrocytes^6,7^. The exact cause of α-syn aggregation is debated, but the importance of this protein in PD is highlighted through genetic studies revealing missense, duplication, or triplication mutations in the SNCA gene encoding α-syn leading to early-onset PD with prominent dementia^8^. Importantly, human patient samples revealed aberrant α-syn distribution peripherally within blood plasma^9^, interstitial fluid^10,11^, and the enteric nervous system (ENS)^12^, implicating prion-like replication and spreading of α-syn. The accumulation of α-syn aggregates can promote sustained activation of pattern-recognition receptors (PRRs), leading to the production of proinflammatory cytokines in microglia^13,14^. These inflammatory mediators from microglia and chemoattractant molecules from damaged neurons possibly play a role in infiltrating peripheral cells into the central nervous system (CNS)^15^. Peripheral inflammation has been shown to exacerbate DA neurodegeneration in numerous animal models of PD^16,17^. Extracellular α-syn aggregates may elicit a self-amplifying cycle of immune responses in the CNS^14,18^ and periphery^19^ through an overproduction of inflammatory mediators, thus providing the tertiary hit required for PD-associated dysfunction to spread to neighboring neurons in the CNS^20,21^ and the periphery^22^. Therefore, immunomodulatory-based approaches aimed at halting the propagation and burden of extracellular α-syn, and in turn diminishing the inflammatory response, are currently being tested as a therapeutic for PD and related synucleinopathies (reviewed in^23^). Recently, it was discovered that natural killer (NK) cells efficiently internalize and degrade α-syn aggregates via the endosomal/lysosomal pathway, a novel and highly relevant function of NK cells in synucleinopathies^24^. The number of circulating NK cells in PD patients is increased compared to non-PD controls^25^. Furthermore, NK cells are present in the human postmortem PD SN^24^ and PD mouse brain^26^, and depletion of NK cells in a mouse model of synucleinopathies is associated with increased neuropathology^24^. In this review, we review α-syn-associated immune responses, the potential role of NK cells, and the mechanisms by which NK cells exert their effects on PD pathogenesis.

## Implications of α-syn-associated immune responses in PD

Although the etiology of PD is considered multifactorial in nature, genetic studies have identified several loci involved in inflammation, including the human leukocyte antigen-DR isotype (HLA-DR), that lead to an increased risk of PD^27^. It is hypothesized that misfolded α-syn may potentiate the observed increase in inflammation seen in PD, as excessive aggregated α-syn can hyperactivate microglia^18^ and promote neurotoxic effects by enhancing the release of tumor necrosis factor-alpha (TNF-α), interleukin (IL) 1-beta (IL-1β), IL-6, nitric oxide (NO), inducible nitric oxide synthase (iNOS), and reactive oxygen species (ROS). Microglia phagocytose extracellular α-syn^28^ via various receptors, including Toll-like receptors^13,14^. As microglia clear α-syn aggregates, they become activated independent of internalization and phagocytosis^29,30^. The distinct mechanisms of α-syn-induced activation seem to be α-syn species-specific^28^. For example, monomeric and mutant α-syn can bind to CD36 on microglia, leading to the production of TNF-α, oxidative stress, and Erk phosphorylation^31,32^, while oligomeric α-syn interacts with CD11b to induce nicotinamide adenine dinucleotide phosphate (NADPH) oxidase and ROS production^21^. The α-syn-induced cascade of proinflammatory cytokines and oxygenating mediators from microglia is sufficient to induce neurodegeneration^33^.

Neuronal expression of α-syn also induces reactive astrogliosis^26,34–36^. Furthermore, α-syn-containing inclusions are present astrocytes in sporadic PD cases^7^ and transgenic mouse models of synucleinopathy^6^. α-Syn is efficiently transferred between astrocytes^37,38^ and from neurons to astrocytes^37^ in vitro and in vivo. Astrocytic transfer of α-syn is conducted via direct contact and tunneling nanotubes (TNTs)^38^. α-Syn is localized to the lysosomal compartment of astrocytes and neurons; however, a progressive increase in cleaved and full-length α-syn was displayed in neurons over time, while astrocytes displayed efficient degradation of α-syn^37^. However, failure of the lysosomal digestion of excess α-syn oligomers in astrocytes results in α-syn deposits in the trans-Golgi network followed by endoplasmic reticulum swelling and mitochondrial disturbances^38^. Moreover, exogenous α-syn induced increased mRNA expression of proinflammatory cytokines (IL-1β, TNF-α), iNOS, and cyclooxygenase-2 (COX-2) from primary mouse astrocytes in a TLR4-dependent manner^39^.

Peripheral inflammation has been deemed a significant contributing factor in PD pathogenesis. Serum levels of IL-1β, IL-2, IL-10, interferon-gamma (IFN-γ), and TNF-α have been correlated with the severity of PD symptoms^40,41^ and rate of disease progression^42^. Serum autoimmune antibodies against α-syn are significantly elevated in PD patients^43^. Autoantibody titers, α-syn monomers, and oligomers plus fibrils in 72%, 56%, and 17% of PD patients are elevated, respectively, within a 5-year disease duration^44^, which implicates systemic adaptive immune responses against different α-syn species. PD patients also have a T helper cell 1 (Th1) bias in peripheral blood with naïve CD4+ T cells from patients preferentially differentiating to a Th1 proinflammatory lineage and showing augmented production of IFN-γ and TNF-α^45^. Moreover, peripheral blood mononuclear cell (PBMC)-derived CD4+ T cells from PD patients specifically react to antigenic major histocompatibility class two (MHC-II) epitopes derived from α-syn^46^, revealing T cell specificity to the α-syn antigen. Substantial increases in inflammatory cytokines (IL-1β, TNF-α, IL-6)^47^ and intestinal permeability^48–50^ are correlated with the presence of α-syn aggregates within the gut^51^. Increased intestinal permeability is thought to result from reductions in barrier-promoting proteins and disruptions of tight-junction networks^49^, a phenotype consistent with low-grade inflammation^52,53^.

## Properties of NK cells

NK cells are bone marrow (BM)-derived hematopoietic cells^54^ that represent 10–15% of total circulating lymphocytes^55^ and are widely located throughout lymphoid and nonlymphoid tissues^56,57^. NK cells primarily target and destroy malignant cells through germline-encoded activating and inhibitory receptors^58^, perforin and granzyme production^59–61^ following immune synapse formation with a target cell, and death receptor pathways Fas Ligand and tumor necrosis factor-related apoptosis-inducing ligand (TRAIL)^62,63^. Immunological synapse formation leads to polarization of NK cells towards their target synapse, followed by lysosomal docking at the plasma membrane and finally the fusion and release of contents^61^. NK cell function extends beyond malignant cell lysis with various roles, such as antimicrobial defense^64,65^, formation of immunological memory^66^, resolution of inflammation^67–70^, and endocytosis of extracellular proteins^24,71^.

**Fig. 1:**
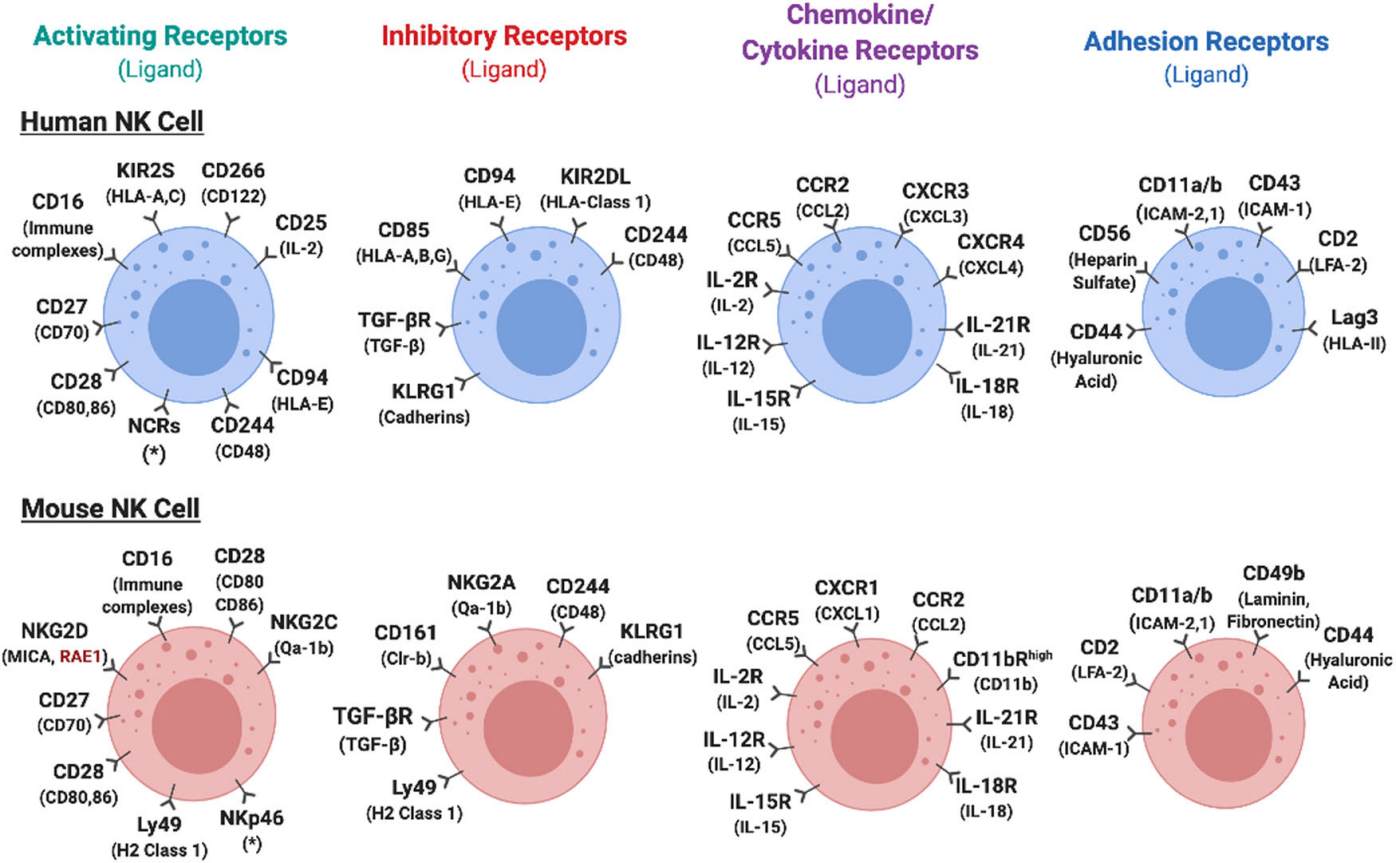
Human and mouse NK cell receptors. NK cell function is mediated by the integration of activating and inhibitory signals. The combinatorial nature of signal integration involves ITAM (immunoreceptor tyrosine-based activation motif)-bearing molecules, ITIMs (immunoreceptor tyrosine-based inhibitory motifs), and other stimulatory receptors for cytokines and adhesion molecules. *Activated by multiple ligands (viral hemagglutinins, unknown tumor ligands).

NK cells are capable of recognizing self- and nonself-molecules through the expression of a variety of activating and inhibitory receptors that regulate NK cell activity (summarized in Fig. 1). NK cell activating and inhibitory receptors contain immunoreceptor tyrosine activating motifs (ITAMs) and immunoreceptor tyrosine-based inhibitory motifs (ITIMs), respectively^58,72,73^. NK cells express killer immunoglobulin-like receptors (KIRs) in humans for molecules known as human leukocyte antigen DR isotype (HLA-DR) and Ly49 receptors in rodents for major histocompatibility complex class I (MHC-I)^72,74,75^. All nucleated cells express HLA-DR/MHC-I molecules, and therefore, the binding of these NK cell receptors to these molecules is the prominent mechanism for ‘tolerance’ of self-cells^76–78^. Other pivotal inhibitory receptors found on NK cells are CD85 for HLA-A, CD94 for HLA-E in humans and NKG2A for Qa-1b and CD244 for CD48 in mice^79^ (Fig. 1). Cells undergoing malignant transformation often downregulate the expression of MHC class I molecules^62^, which permit the activation of NK cells. Activating receptors found on the surface of NK cells in humans include CD16 for immune complexes, CD122 for IL-2 and IL-15, CD266 (DNAM-1) for Nectin-2, and KIR2S for HLA-1 (Fig. 1). Activating receptors on NK cells found in mice include CD16 for immune complexes, NKG2C for Qa-1b, NKG2D for MHC class I polypeptide-related sequence A (MICA), and CD28 for CD80 (Fig. 1) (summarized in^79^).

In humans, NK cells are primarily divided into CD3- CD16+ CD56^dim^ and CD3- CD16+ CD56^bright^ subsets^56^. Approximately 90% of peripheral blood and splenic NK cells are CD3-CD16+ CD56^dim^, which have cytotoxic effector functions, including the production of perforin and IFN-γ, upon interaction with tumor cells^80^. CD3- CD16+ CD56^bright^ NK cell subsets play an immunoregulatory role through cytokine production that can directly and indirectly modulate innate and adaptive immune responses^81^. In the CNS, the majority of NK cells in cerebrospinal fluid (CSF) are CD56^bright^ NK cells^82^. Both CD3- CD16+ CD56^dim^ and CD3- CD16+ CD56^bright^ NK cell subsets can take on highly specific effector functions through dynamic and combinatorial alterations in receptor expression under pathophysiological conditions such as viral infections and autoimmune diseases^83,84^. In mice, NK cells are generally defined as CD3-NK1.1+ or CD3-NKCR1/NKp46+ cells that typically express integrin alpha 2/CD49b, integrin alpha M/CD11b, CD27, T-bet, and Eomes and lack expression of CD127/IL-7R alpha^79,85^. Three major subsets of mouse NK cells have been characterized based on the differential expression of integrin alpha M/CD11b and CD27, including CD11b^dim^ CD27^bright^ NK cells, CD11b^bright^CD27^dim^ NK cells, and CD11b^bright^CD27^bright^ NK cells. CD27^dim^ NK cells have lower cytotoxic potential and produce lower levels of cytokines than CD27^bright^ NK cells^86^. However, strain differences have been observed, as C57BL/6J mouse NK cells are identified by the presence of NK1.1 (NKR-P1C) and NCR1 (NKp46/CD335), while CD49b (DX5, Integrin VLA-2α) is a common NK cell marker in other mouse backgrounds^86^.

## The presence of NK cells in the CNS

The existence of brain residential NK cells has been strongly supported by transcriptomic analysis of brain myeloid cells, revealing that NK cells are identified as small but distinct and biologically meaningful clusters of cells in the brain parenchyma^87,88^. Peripheral NK cells are recruited to the CNS during high levels of inflammation by chemokines such as CX3C ligand 1 (CX3CL1) produced by neurons^89^ or C-C motif chemokine ligand 2 (CCL2) and CXC ligand 10 (CXCL10) produced by microglia, astrocytes, and other inflammatory cells^57^. NK cells found in the CNS display various functions in neurological disorders. NK cells migrate to the mouse brain in a CX3CL1-dependent manner in the experimental autoimmune encephalomyelitis (EAE) model of multiple sclerosis (MS)^69^ and attenuate inflammation in the early stages of EAE, suggesting anti-inflammatory effects^57^, while others reported neurotoxic effects in EAE^90^. NK cells can inhibit microglial transactivation of T helper 17 cell (Th17) signature transcription factors on pathogenic T cells and in turn decrease neuroinflammation^69^. Furthermore, NK cells display cytotoxicity against astrocytes^91^ during infection through astrocytic expression of the inducible NKp44 ligand^92^. NK cells were found in the motor cortex and spinal cord in a CCL2-dependent manner and modulate the progression of motor neuron degeneration in amyotrophic lateral sclerosis (ALS)^93^. In neurological autoimmune conditions, NK cells have been shown to reciprocally interact with neural stem cells (NSCs) to regulate neural repair during the chronic stage of disease progression^94^, demonstrating the capacity to engage with neural cells.

## Implications of NK cells in PD pathologies

**Fig. 2:**
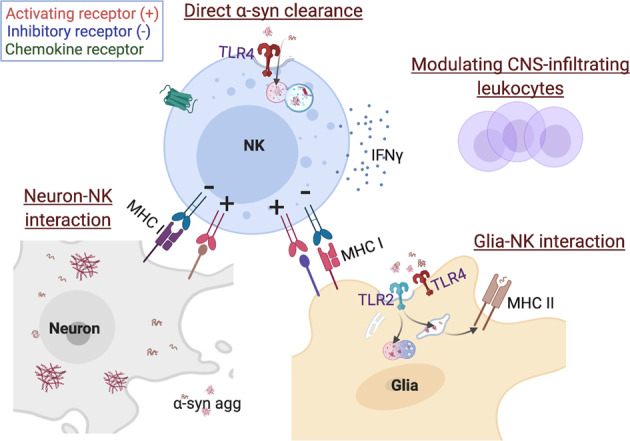
Schematic representation of the potential mechanisms of NK cells in the CNS. The proposed mechanisms of NK cells in the CNS are as follows: 1) NK cells efficiently scavenge α-syn aggregates via receptor-mediated endocytosis; 2) NK cells modulate CNS inflammation by directly interacting with neurons or glia. NK cells have been involved in the cytotoxicity to immature or damaged neurons. Neurons and microglia may change the surface expression of ligands for the inhibitory receptor of NK cells or ligands for activating receptors that affect the activation status of NK cells; 3) Lastly, NK cells may attenuate neuroinflammation by modulating CNS-infiltrated leukocytes.

In PD patients, NK cell numbers are increased in the blood compared to controls^25,95,96^. Mihara et al reported that NKG2A expression on circulating NK cells is lower than that of non-PD controls with no changes in NKG2D expression^95^. Another study reported that the percentages of NKG2D-positive NK cells were higher in PD patients^25^. The functional activity of NK cells against K562 leukemia cells is positively correlated with disease duration in PD patients, suggesting that NK activity increases as the disease advances^95^. However, these studies must be further evaluated, as K562 cells express high levels of ligands for NKG2D but very low levels of ligands for NKG2A (HLA-E).^97^. Although these findings are purely correlational, they do suggest a consistent deviation in NK cell number and receptor expression patterns during pathogenesis^96,98,99^. In addition, analysis of subsets of NK cells and their function associated with PD pathologies needs to be conducted to further understand their function in PD. The presence of NK cells in human brains with synucleinopathies was recently reported^24^. Immunohistochemical analysis of postmortem brain tissue containing abnormal α-syn aggregates, including PD, DLB, Parkinson’s disease dementia (PDD), and progressive supranuclear palsy (PSS) cases, demonstrated that NK cells were found in close proximity to α-syn aggregates^24^. Moreover, NK cells are also present in the brain parenchyma of mouse models of PD^24,26^. While the presence of NK cells in the CNS parenchyma is undoubtedly significant, the role NK cells play in PD pathogenesis has remained elusive until a recent study suggested a neuroprotective role of NK cells in PD^24^. The study demonstrated that NK cells reduce synuclein burden in vitro, and systemic depletion of NK cells in a preclinical mouse model of PD results in increased pathological α-syn burden in numerous brain regions, including the striatum, SNpc, and brainstem^24^. The study also demonstrated that increased α-syn pathology is positively correlated with gross motor deficits and mortality^24^. Furthermore, NK cell-deficient animals displayed increased inflammation in the CNS, as shown through glial fibrillary acidic protein (GFAP) and ionized calcium binding adaptor molecule-1 (Iba-1) immunoreactivity^24^. While this study provides a critical foundation for the argument that NK cells play a protective role in PD and potentially other synucleinopathies, the precise mechanism by which NK cells exert protection requires further investigation. The possible mechanisms by which NK cells exert effects during PD pathogenesis (α-syn deposition, augmented inflammation, DA neurodegeneration, and motor dysfunction) are as follows (summarized in Fig. 2).

### NK cells as direct scavengers of α-syn species

α-Syn deposition has been shown to promote the release of senescence-associated secretory (SASP) profiles from cells^100^, which converts senescent cells into continuous sources of proinflammatory mediators, reactive oxygen species and metalloproteinases^101,102^. NK cells can recognize and clear senescent cells through a mechanism involving perforin and granzyme-containing granule exocytosis and production of IFN-γ following senescent cell interaction^103^ Therefore, NK cells may target senescent cells burdened with α-syn for lysis. Furthermore, human NK cells efficiently scavenge various forms of α-syn species^24^, making it plausible that NK cells can reduce the α-syn burden seen in PD patients. Although NK cells are not classically defined as phagocytic cells, they possess efficient endocytosis machinery, as receptor trafficking through endocytic pathways has been well documented in NK cells^71^. α-Syn aggregates are degraded within NK cells, and cytoplasmic α-syn is colocalized with endosomal and lysosomal protein markers^24^. Importantly, α-syn aggregates attenuate NK cell release of IFN-γ^24^. This finding is highly significant, as α-syn augments the release of proinflammatory cytokines and oxidative species in microglia during the phagocytosis of α-syn aggregates^33^. It is also conceivable that NK cells will preferentially home to areas of increased synuclein burden.

### Interaction of NK cells and CNS-resident cells in PD

NK cells have previously been shown to be selectively involved in neurotoxicity in immature or injured neurons via an NKG2D-retinoic acid early inducible gene 1 (RAE-1)-mediated mechanism following peripheral nerve injury, leading to the resolution of painful neuropathies^104,105^. NKG2D ligands comprise several MHC class I-like molecules^106^. From analysis of mRNA-seq data by Dumitriu et al.^107^, the levels of stress-inducible glycoproteins MICA and MICB were significantly higher in PD brains than in healthy brains, which could induce interaction with NK cells within the CNS. Thus, NK cells may selectively target neurons with α-syn inclusions via NKG2D ligands. Another potential mechanism by which NK cells exert protection is through a microglia-NK cell interaction. This interaction can induce decreased expression of the MHC class I molecule Qa1 on activated microglia, which in turn triggers NK cell-mediated cytotoxicity towards hyperactive microglia^69^. As microglia are known to become aberrantly activated in the presence of sustained α-syn burden, targeting microglial activation states by suppressing their deleterious proinflammatory neurotoxicity may be a valid therapeutic approach for PD treatment^23^. Therefore, NK cells could exert protection by mitigating microglial toxic effects within the CNS.

### Interaction of NK cells and immune cells and their implications in PD

Systemic NK cell depletion in vivo in EAE led to disease enhancement associated with increased autoreactive T cell proliferation and a proinflammatory response^108^. NK cells display enhanced cytotoxicity towards the myelin proteolipid protein (PLP) autoreactive T cell line in comparison to naïve splenic T cells in vitro^90^, indicating that NK cells exert protection in EAE by killing myelin antigen-specific T cells. SN DA neurons display MHC-I in response to microglia stimulated with α-syn and take on antigen-presenting cell (APC) functions such as loading and displaying of antigen^46^. Neuronal antigen-loaded MHC-I was competent to trigger cytotoxic T cell (CTL)-mediated neuronal death in vitro^46^. It seems plausible that T cells are autoreactive to α-syn, as peptides derived from α-syn elicit in vitro responses from CD8+ and CD4+ T cells in PD patients but not healthy controls^109^. Therefore, NK cells could exert effects through direct and indirect interactions with T cells. NK cells can negatively regulate the T cell response directly by their ability to recognize and kill activated T cells^110–112^. In vitro experiments show that activated CD4+ and CD8+ T cells are susceptible to NK cell-mediated lysis through perforin-dependent mechanisms^110^ and increased expression of NKG2D ligands^112^. Of high relevance, in vivo activated murine T cells become targets for NK cells under LPS treatment, which mimics an inflammatory condition^110^.

### NK cells are a major source of IFN-γ and their potential role in PD

Many studies have demonstrated a relationship between PD and IFN-γ levels, with recent evidence showing elevated blood plasma levels of IFN-γ in PD patients^113^. NK cells are major producers of IFN-γ^114^, and NK cell depletion in a murine model of synucleinopathy led to a six-fold reduction in IFN-γ serum concentration^24^. IFN-γ directly or indirectly affects APCs by increasing the expression of subunits of MHC class I and II molecules, transporters associated with antigen processing (TAP1/2), invariant chain, and the expression and activity of the proteasome^115^. IFN-γ contributes to macrophage activation by increasing phagocytosis and priming the production of proinflammatory mediators^115^. Furthermore, Th1 development is heavily influenced by IFN-γ produced by NK cells^115^.

In the CNS, IFN-γ ameliorated autoimmune disease symptoms with EAE^116,117^, implicating a neuroprotective effect. Furthermore, a study showed the potential role of α-syn in modulating the expression of IFN genes, as IFN genes were downregulated in the CNS following brain infection in α-syn knockout mice^118^. Whether IFN-γ potentiates or attenuates the phagocytic activity of microglial α-syn aggregates needs to be investigated. α-Syn-containing inclusions are also present in the lysosomal compartments of astrocytes in sporadic PD cases^7^ and transgenic mouse models of α-synucleinopathy^6^. Microarray analysis of IFN-γ response genes in astrocytes showed increased expression of genes associated with protein degradation (Ubiquitin D) and proteasome degradation (proteasome subunit β9)^119^, implicating that IFN-γ may promote the lysosomal digestion of excess α-syn and reduce organelle dysfunction. As the amount of IFN-γ produced by NK cells decreases in older adults^120,121^, it could have deleterious effects on the ability of APCs to manage pathogens such as α-syn aggregates centrally and peripherally.

### Role of NK cells in the gut and their potential role in PD pathologies

Peripherally, gut-resident NK cells are found as intraepithelial lymphocytes (IEPs) and in the lamina propria^122^. The cytokines produced by NK cells, including IFN-γ and TNF, have a primary role in gut homeostasis and damage (reviewed in ref. ^122^). In addition, NK cells play key roles against gut infections of *Salmonella*, *Listeria*, *Citrobacter rodentium*, and other enteric pathogen injections in mice^123–127^. NK cell activity in the gut with autoimmune inflammatory bowel diseases (IBDs), such as Crohn’s disease and ulcerative colitis, is substantially dampened (reviewed in ref. ^122^). Macrophages within the myenteric plexus are present at sites of aggregated α-syn deposition and are able to phagocytose deposits, as evidenced by inclusions present in the cytoplasm of macrophages^51^. As α-syn inclusions are found within the ENS in PD, they may mobilize resident macrophage populations to clear this protein and induce an inflammatory milieu within the gut. NK cell-macrophage interactions have been characterized via LPS stimulation of macrophages, inducing NKG2D upregulation and subsequent lysis of activated macrophages by NK cells^128^. Therefore, gut-resident NK cells, which come into contact with a multitude of antigens and adaptive immune cells (macrophages, dendritic cells, T cells)^122^, may have the capacity to mitigate the peripheral macrophage proinflammatory response to the α-syn antigen.

### NK cell immunosen*e*scence and its potential role in PD pathologies

NK cell immunosenescence can impair crosstalk between the innate and adaptive immune systems^129^, which may have substantial implications for the aging population. Human studies have shown significant increases in the frequency and total number of circulating NK cell populations with age^129–131^, while there are significant reductions in both the numbers and percentage of splenic NK cells in aged mice (unpublished observation). Since human studies are restricted to circulating mononuclear cells (PBMCs), the profiles of lymphocytes with aging in local tissues may explain the observed differences between species. There have been conflicting reports regarding the functionality of aging NK cells, as human NK cells are hyporesponsive or hyperresponsive to IL-2 compared to young NK cells as measured by IFN-γ production^120,121,132^. As IFN-γ function is so diverse, the potential implications for dysregulated release of this proinflammatory mediator with age are immense.

## Conclusion and future directions

The mechanisms by which NK cells can exert protection in synucleinopathies are abundant. NK cells finely orchestrate immune responses and modulate inflammation, making them a highly relevant cell type to study in inflammatory conditions. NK cells may play a key role in alleviating a sustained, unmitigated immune response to α-syn systemically. Furthermore, NK cells can internalize and degrade α-syn aggregates and subsequently reduce the α-syn burden. However, additional studies must be conducted to elucidate the precise mechanisms by which NK cells exert effects in PD. In addition, NK cell immunosenescence may result in the accumulation of α-syn aggregates and the deceleration of the resolution of immune responses. As a future direction, age-related alterations in NK cell phenotypes would provide insight into the relationship between NK cell immunosenescence and age-related neurological disorders. In addition, PD has an increased prevalence in males compared to females; therefore, interrogating the effects of sex on NK cell phenotype would further expand our knowledge on how NK cells contribute to age-related neurodegenerative diseases that display sexual dimorphic patterns.
